# Bioimpedance-derived compartmental fluid status and prognosis in chronic heart failure

**DOI:** 10.1093/eschf/xvag002

**Published:** 2026-01-22

**Authors:** Jorge Montiel, Alicia Lucas, Pablo Peiró, Gonzalo Núñez, Miguel Lorenzo, Andrea Gasull, Gema Miñana, Anna Mollar, Enrique Santas, José Luis Górriz, Juan Sanchis, Julio Núñez, Rafael de la Espriella

**Affiliations:** Cardiology Department, Hospital Clínico Universitario de Valencia, Avda. Blasco Ibáñez 17, Valencia 46010, Spain; Internal Medicine Department, Hospital Clínico Universitario de Valencia, Valencia, Spain; Department of Medicine, Universitat de València, Valencia, Spain; Cardiology Department, Hospital Clínico Universitario de Valencia, Avda. Blasco Ibáñez 17, Valencia 46010, Spain; INCLIVA Biomedical Research Institute, Valencia, Spain; Cardiology Department, Hospital Clínico Universitario de Valencia, Avda. Blasco Ibáñez 17, Valencia 46010, Spain; INCLIVA Biomedical Research Institute, Valencia, Spain; Cardiology Department, Hospital Clínico Universitario de Valencia, Avda. Blasco Ibáñez 17, Valencia 46010, Spain; Cardiology Department, Hospital Clínico Universitario de Valencia, Avda. Blasco Ibáñez 17, Valencia 46010, Spain; Department of Medicine, Universitat de València, Valencia, Spain; INCLIVA Biomedical Research Institute, Valencia, Spain; Cardiology Department, Hospital Clínico Universitario de Valencia, Avda. Blasco Ibáñez 17, Valencia 46010, Spain; INCLIVA Biomedical Research Institute, Valencia, Spain; Centro de Investigación Biomédica en Red—Cardiovascular (CIBER-CV), Madrid, Spain; Cardiology Department, Hospital Clínico Universitario de Valencia, Avda. Blasco Ibáñez 17, Valencia 46010, Spain; Department of Medicine, Universitat de València, Valencia, Spain; INCLIVA Biomedical Research Institute, Valencia, Spain; Department of Medicine, Universitat de València, Valencia, Spain; INCLIVA Biomedical Research Institute, Valencia, Spain; Nephrology Department, Hospital Clínico Universitario de Valencia, Valencia, Spain; Cardiology Department, Hospital Clínico Universitario de Valencia, Avda. Blasco Ibáñez 17, Valencia 46010, Spain; Department of Medicine, Universitat de València, Valencia, Spain; INCLIVA Biomedical Research Institute, Valencia, Spain; Centro de Investigación Biomédica en Red—Cardiovascular (CIBER-CV), Madrid, Spain; Cardiology Department, Hospital Clínico Universitario de Valencia, Avda. Blasco Ibáñez 17, Valencia 46010, Spain; Department of Medicine, Universitat de València, Valencia, Spain; INCLIVA Biomedical Research Institute, Valencia, Spain; Centro de Investigación Biomédica en Red—Cardiovascular (CIBER-CV), Madrid, Spain; Cardiology Department, Hospital Clínico Universitario de Valencia, Avda. Blasco Ibáñez 17, Valencia 46010, Spain; INCLIVA Biomedical Research Institute, Valencia, Spain; Centro de Investigación Biomédica en Red—Cardiovascular (CIBER-CV), Madrid, Spain

**Keywords:** Chronic heart failure, Bioimpedance analysis, Congestion

## Abstract

**Introduction:**

Novel bioimpedance analysis (BIA) devices may improve the precision of fluid overload assessment, potentially offering additional prognostic information beyond clinical parameters in patients with HF. This study aimed to evaluate the association between fluid overload indexes obtained with a novel bioelectrical impedance device (BioScan touch i8 IVF, MALTRON®) and the risk of cardiovascular events in ambulatory patients with HF.

**Methods:**

Volume excess was assessed using BioScan Touch i8 IVF (MALTRON), which differentiates fluid distribution across body compartments. Volume-excess variables (total, intravascular, and tissue) were analysed both as quartiles (Q) and as continuous measures. Their independent associations with the composite of all-cause death or worsening HF were adjusted for established prognostic markers, including a clinical congestion score (CCS) and N-terminal pro-B-type natriuretic peptide.

**Results:**

Among 386 ambulatory patients with stage C chronic HF, median total, intravascular, and tissue fluid excess were 1.3 L (Q1–Q3: 0.5, 2.3), 0.1 L (Q1–Q3: −0.1, 0.4), and 1.9 L (Q1–Q3: 1.1, 2.8), respectively. Total and tissue fluid excess had the strongest correlation with the CCS (Total fluid excess rho = 0.31; *P* < .001, tissue fluid excess rho = 0.33; *P* < .001). In adjusted analyses, patients in the highest quartile had significantly higher risk of the composite outcome compared with Q1: total fluid excess hazard ratio (HR) 2.11 [95% confidence interval (CI) 1.21–4.05, *P* = .010], intravascular fluid excess HR 2.16 (1.19–3.90, *P* = .011), and tissue fluid excess HR 2.81 (1.49–5.32, *P* = .001).

**Conclusion:**

Compartment-specific estimates of fluid overload obtained with the BioScan Touch i8 IVF were independently associated with adverse outcomes in ambulatory patients with chronic HF, beyond NT-proBNP and clinical surrogates of congestion. These findings support the potential role of BIA-derived indexes, particularly tissue fluid excess, as complementary tools for risk stratification in this population.

## Graphical Abstract

**Graphical Abstract:**
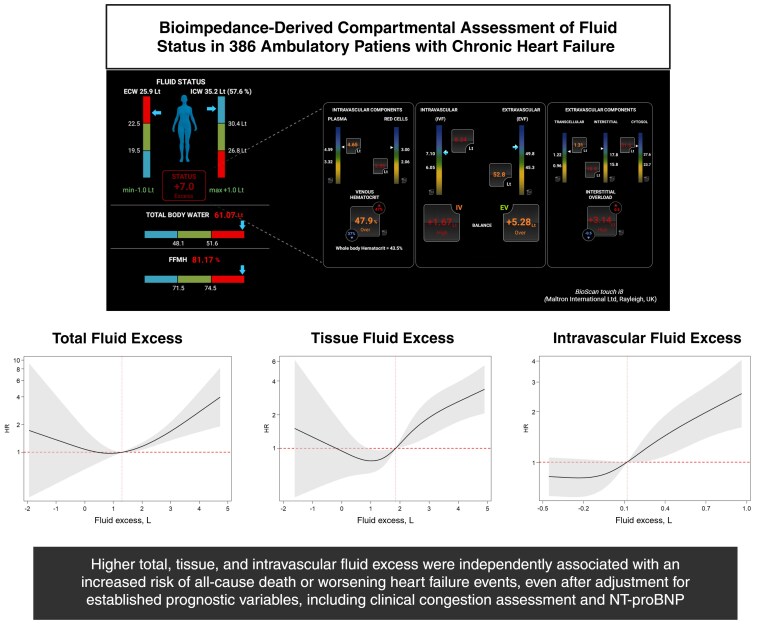
Bioimpedance-derived compartmental assessment of fluid status in chronic heart failure.

## Introduction

Congestion plays a central role in the pathophysiology, clinical status, and prognosis of heart failure (HF).^1^ Yet, many apparently stable outpatients without overt clinical signs and symptoms of congestion might have subclinical fluid overload, leading to diagnostic uncertainty and potential delays in treatment. Traditional physical examination has variable sensitivity and specificity, and natriuretic peptides—although widely used—are influenced by age, body mass index (BMI), renal function, and atrial fibrillation, limiting their accuracy for detecting true volume overload.^2–5^ Similarly, although targeted ultrasound evaluation of extra-cardiac structures (e.g. lung tissue, venous system, kidneys) can potentially improve volume assessment and guide management,^3,6–8^ these techniques remain constrained by equipment availability, operator dependency, measurement variability, and limited validation across care settings.

Bioimpedance technology is non-invasive, broadly accessible, and relatively inexpensive. It has been widely applied in nephrology (particularly in haemodialysis patients) to assess volume status with minimal operator variability.^9^ Despite these advantages, most bioimpedance analysis (BIA) devices cannot reliably distinguish fluid distribution across different body compartments. This limitation stems from the underlying assumptions of uniform fluid distribution, which may not be valid in HF.^10^

This study aimed to evaluate the association between fluid overload indexes obtained with a novel bioelectrical impedance device (BioScan touch i8 IVF, MALTRON®) and the risk of cardiovascular events in ambulatory patients with HF.

## Methods

### Study design and population

Patients were prospectively enrolled in a single tertiary HF clinic between July 2022 and December 2023. Subjects were eligible if they were >18 years old, provided written informed consent, and had a confirmed history of symptomatic chronic HF according to European Society of Cardiology (ESC) HF guidelines (7). Patients were included during routine follow-up visits, excluding those needing intravenous diuretics for worsening HF symptoms on the day of enrolment. All study participants were previously followed up in a specialized HF clinic with readily accessible outpatient care (day-hospital setting) and with frequent clinical and laboratory monitoring [including a complete blood count, serum electrolytes, blood urea nitrogen, serum creatinine, and N-terminal pro-B-type natriuretic peptide (NT-proBNP)]. Scheduled laboratory tests are commonly drawn within one week before the next scheduled visit in order to ensure laboratory results are forwarded to HF clinicians in a timely manner. The study complied with the Declaration of Helsinki and was approved by the local institutional review committees. All patients provided written informed consent.

### Baseline data collection

Standardized baseline evaluation consisted of an assessment of medical history [sex, age, cardiovascular risk factors, comorbidities, New York Heart Association (NYHA) functional class, physical examination (blood pressure, heart rate, weight, height, and BMI), and current pharmacological treatment]. Medication data captured use of beta-blockers, angiotensin-converting enzyme inhibitors, angiotensin receptor blockers, angiotensin receptor-neprilysin inhibitors, mineralocorticoid receptor antagonists, sodium–glucose cotransporter 2 inhibitors (SGLT2i), and diuretic therapy (loop- and thiazide/thiazide-like diuretics).

Congestion was assessed by evaluating orthopnoea, jugular venous pressure (JVP), rales, and pedal oedema using a two-point scale (0 = absent, 1 = present). A composite congestion score (CCS) was then calculated by summing the individual scores for orthopnoea, JVP, rales, and pedal oedema, yielding a total score ranging from 0 to 4. In addition, standard laboratory data [including a complete blood count, serum electrolytes, blood urea nitrogen, serum creatinine, glomerular filtration rate (calculated by CKD-EPI), and N-terminal pro-B-type natriuretic peptide (NT-proBNP)] obtained within the last week (as per routine laboratory monitoring), and echocardiographic data (left ventricular ejection fraction (LVEF) and tricuspid annular plane systolic excursion), obtained within the last 12 months before inclusion were also registered.

All samples for biomarker analysis were collected and processed by the local laboratory according to standardized conditions and protocols.

### Bioelectrical impedance analysis

Bioelectrical impedance measurements were performed with the BioScan touch i8 IVF device (Maltron International Ltd, Rayleigh, UK) with the patient in a supine position. Electrodes were attached to the patient’s right and left hand (one electrode at the metacarpal and one electrode at the carpal region) and right and left foot (one electrode at the metatarsal region and one electrode at the talus). Measurements were performed by HF nurses not involved in the patient interview or physical examination according to the manufacturer’s instructions.

Unlike traditional BIA devices, which estimate total body water, the BioScan touch i8 IVF uses novel technologies to measure tissue-specific properties. These data are processed collectively through proprietary algorithms that account for tissue conductivity, ion concentration, and cellular structure, fluid distribution, and physiological factors.

Intravascular, extravascular, and tissue fluid excess were calculated using individualized target ranges provided by the BioScan touch i8 IVF. Specifically, the target intravascular, extravascular, and tissue fluid values were calculated as the averages of their respective upper and lower limits. To quantify fluid excess across body compartments, the actual measured value for each compartment was subtracted from its respective mid target value.

### Treatment

The treating physicians were blinded from the bioelectrical impedance measurements. Consequently, pharmacological treatment was individualized based on clinical judgment in line with established HF guidelines.

### Clinical endpoints

The endpoint of interest was time to the composite of death or worsening HF at one year and its components. An episode of worsening heart failure (WHF) was either an unplanned HF hospitalization or an urgent HF visit requiring parenteral diuretic therapy. WHF events were prospectively standardized across the cohort using pre-specified definitions.^11^  *HF hospitalization* was defined as events requiring an overnight stay accompanied by signs and symptoms of HF and receipt of intravenous HF therapies (diuretics, vasodilators, inotropes). *Urgent HF visits* were defined as receiving parenteral diuretic therapy during urgent, unscheduled ambulatory clinic or emergency department visits without formal inpatient hospitalization requiring an overnight stay. Outcome assessment was performed by systematically verifying patient survival status and hospital readmissions using electronic medical records from the Valencian Community public healthcare system. Data was retrieved from the SIA-GAIA and Orion Clinic databases, which comprehensively document all healthcare encounters within the regional network. Event adjudication was conducted by site investigators blinded to the exposure variable.

### Statistical analysis

Baseline characteristics among bioelectrical impedance fluid measurements (total, intravascular, and tissue fluid excess) quartiles (Q1–Q4) are presented as frequencies and percentages for categorical variables, mean ± SD, or median (Q1–Q3) for continuous variables. A nonparametric test for trend across groups, an extension of the Wilcoxon rank sum test, was used to examine for variation in continuous baseline characteristics across BIA quartiles.

Spearman correlation was determined between total, intravascular, and tissue fluid excess and age, systolic blood pressure, serum sodium, estimated glomerular filtration rate, NT-proBNP, and CCS.

For survival analysis, a composite event variable was created that included all-cause death, HF hospitalization, or an urgent HF visit requiring parenteral diuretic therapy. Incidence rates for each outcome of interest according to BIA fluid measurements are presented per 100 person-years of follow-up and are presented graphically using Kaplan–Meier survival curves. The relationship between total, intravascular, and tissue fluid excess at baseline and the subsequent outcome was analysed using multivariable Cox proportional hazards regression models or Fine and Gray regression model accounting for all-cause mortality as a competing event as appropriate. Risk estimates for the Cox and the Fine and Gray analyses were expressed as hazard or sub-distribution hazard ratios (HRs), respectively, with their 95% confidence intervals (95% CIs). Candidate covariates were chosen based on prior medical knowledge/biological plausibility, independent of their *P*-values. Covariates included in the final multivariable model for the composite endpoint were age, CCS ≥2, log-transformed NT-proBNP, blood urea nitrogen, plasma sodium, treatment with renin-angiotensin-system inhibitors, aldosterone receptor blockers, and SGLT2 receptor inhibitors. The proportionality assumption of the Cox proportional hazards model was assessed using Schoenfeld residuals.

Due to the skewed distribution of BIA-derived fluid measurements, the relationships between total, tissue, and intravascular fluid excess as continuous variables and outcomes were analysed using restricted cubic splines with four knots at the 5th, 35th, 65th, and 95th percentiles, using the median value of each variable as the reference.

The predictive value of BIA-derived fluid measurements and their additional predictive value to the CCS were examined using the area under the curve (AUC) from receiver-operating characteristic curves following logistic regression models. The additional predictive value was expressed using a continuous net reclassification index (NRI) and integrated discrimination improvement metric with 95% CIs.

We set a two-sided *P*-value of <.05 as the threshold for statistical significance. Stata 18.5 [Stata Statistical Software, Release 15 (2017); StataCorp LP, College Station, TX, USA) was used for this analysis. Correlation heatmaps were implemented in R version 4.4.2 (R Foundation for Statistical Computing, Vienna, Austria).

## Results

The mean age of the sample was 76 ± 11 years, 176 (46%) were women, 50 (13%) were on NYHA class III–IV, and 229 (59%) had a LVEF ≥50%. The median (p25, p75%) of NT-proBNP was 1009 pg/ml (428, 2390), and 228 (59%) patients had at least one clinical sign of congestion at the moment of BIA analysis.

The median (p25, p75%) total, intravascular, and tissue volume excess was 1.3 L (0.5, 2.3), 0.1 L (−0.1, 0.4), and 1.9 L (1.1, 2.8), respectively. Baseline characteristics across quartiles of total fluid excess are shown in *Table 1*. Patients with higher total fluid excess tended to be older, have worse NYHA class, and received more use of loop or thiazide diuretics. Likewise, patients in the upper quartiles exhibited higher NT-proBNP values and had greater evidence of clinical congestion.

**Table 1:** Baseline characteristics across quartiles of total fluid status

Variable	Quartiles of total fluid status				
	Q1 (*N* = 98; 25.5%)	Q2 (*N* = 104; 27.0%)	Q3 (*N* = 88; 22.9%)	Q4 (*N* = 95; 24.7%)	*P*-value
Demographics and medical history					
Age, years	73 (65, 81)	76 (70, 82)	78.5 (71.5, 84.5)	78 (71, 84)	<.001
Female, *n* (%)	47 (48.0)	54 (51.9)	43 (48.9)	32 (33.7)	.169
Previous HF admissions, *n* (%)	56 (57.1)	61 (58.7)	58 (65.9)	48 (50.5)	.125
Years since HF diagnosis	2 (1, 4)	2 (1, 4)	3 (1, 5)	2 (1, 5)	.516
Hypertension, *n* (%)	75 (76.5)	85 (81.7)	77 (87.5)	77 (81.1)	.277
Diabetes, *n* (%)	39 (39.8)	39 (37.5)	45 (51.1)	48 (50.5)	.402
IHD, *n* (%)	38 (38.8)	34 (32.7)	49 (55.7)	29 (30.5)	.002
CKD, *n* (%)	25 (25.5)	26 (25.0)	29 (33.0)	37 (38.9)	.681
Maggic risk score	21 (15, 25)	21 (16, 26)	23 (18, 28)	25 (21, 29)	<.001
Physical examination findings					
SBP, mmHg	128 (116, 141)	127 (114, 141)	130 (115, 145)	128 (113, 141)	.926
Heart rate, b.p.m.	73 (64, 82)	72 (65, 82)	72 (61, 79)	70 (61, 80)	.473
NYHA class					<.001
I	19 (19.6)	8 (7.8)	7 (7.9)	8 (8.5)	
II	69 (71.1)	86 (83.5)	73 (83.0)	62 (66.0)	
III	9 (9.3)	9 (8.7)	7 (8.0)	22 (23.4)	
IV	0 (0.0)	0 (0.0)	1 (1.1)	2 (2.1)	
Peripheral oedema, *n* (%)	9 (9.2)	9 (8.7)	21 (23.9)	29 (30.5)	<.001
Pulmonary rales, *n* (%)	8 (8.2)	18 (17.3)	13 (14.8)	39 (41.1)	<.001
Orthopnoea, *n* (%)	27 (27.6)	32 (30.8)	27 (30.7)	46 (48.4)	.003
Jugular engorgement, *n* (%)	18 (18.4)	30 (28.8)	38 (43.2)	47 (49.5)	<.001
Clinical congestion score	0 (0, 1)	1 (0, 1)	1 (0, 2)	2 (1, 3)	<.001
Bioimpedance parameters					
Height, cm	167 (160, 170)	163 (157, 172)	167 (160, 175)	170 (159, 175)	.214
Weight, kg	76.2 (67.0, 87.3)	78.0 (68.0, 88.5)	76.9 (67.1, 88.2)	76.5 (67.3, 87.4)	.810
Dry weight, kg	76.5 (66.9, 85.9)	77.1 (66.6, 87.6)	75.0 (65.7, 86.9)	73.0 (62.8, 83.1)	.131
BMI, kg/m^2^	27.6 (25.0, 30.4)	29.4 (25.9, 31.7)	27.5 (24.2, 32.4)	26.7 (24.0, 30.4)	.332
Body fat, %	36.2 (31.6, 40.2)	37.3 (33.1, 42.6)	35.3 (29.8, 41.4)	32.8 (27.0, 39.6)	.006
Phase angle	7.2 (6.5, 7.8)	6.6 (6.2, 7.4)	6.4 (6.0, 7.1)	6.1 (5.7, 6.7)	<.001
Extracellular water, l	14.7 (13.2, 16.8)	15.5 (14.0, 18.2)	16.9 (14.5, 18.8)	18.4 (16.2, 20.5)	<.001
Intracellular water, l	19.9 (17.6, 23.3)	19.5 (17.0, 23.4)	21.0 (17.3, 23.6)	21.4 (18.5, 23.5)	.194
Total fluid excess, l	0.0 (−0.5, 0.4)	1.0 (0.6, 1.4)	1.5 (1.2, 2.3)	3.0 (2.1, 3.8)	<.001
Tissue fluid excess, l	0.7 (0.2, 1.2)	1.7 (1.3, 2.1)	2.1 (1.7, 2.8)	3.1 (2.6, 3.9)	<.001
Intravascular fluid excess, l	−0.1 (−0.3, 0.0)	0.1 (0.0, 0.3)	0.2 (0.0, 0.4)	0.5 (0.2, 0.7)	<.001
Laboratory					
eGFR, ml/min/1.73 m^2^ (CKD-EPI)	56.5 (42.0, 78.4)	53.6 (38.5, 70.0)	51.4 (39.7, 67.4)	51.9 (36.0, 71.1)	.111
Urea, mg/dl	48 (38, 64)	54 (41, 72)	56 (44, 70)	54 (44, 77)	.277
Serum sodium, mEq/l	139 (138, 141)	140 (139, 142)	140 (138, 143)	140 (138, 142)	.239
Haemoglobin, g/dl	13.9 (12.9, 15.4)	13.9 (12.3, 15.4)	13.8 (12.6, 14.7)	13.2 (11.6, 14.4)	<.001
NT-proBNP, pg/ml	721 (204, 1791.0)	699 (392, 1863)	1056 (522, 2319)	1483 (962, 3846)	<.001
Echocardiography					
LVEF, %	51 (40, 60)	57 (40, 62)	54 (40, 61)	56 (40, 65)	.313
TAPSE, mm	20 (17, 22)	20 (17, 22)	20 (17, 24)	18 (16, 22)	.975
Medical treatment					
RASi, *n* (%)	76 (77.6)	68 (65.4)	70 (79.5)	59 (62.1)	.019
Beta-blockers, *n* (%)	77 (78.6)	86 (82.7)	68 (77.3)	67 (70.5)	.118
MRA, *n* (%)	60 (61.2)	59 (56.7)	44 (50.0)	56 (58.9)	.454
SGLT2i, *n* (%)	67 (68.4)	66 (63.5)	64 (72.7)	66 (69.5)	.556
Loop diuretics, *n* (%)	66 (67.3)	84 (80.8)	68 (77.3)	80 (84.2)	.013
Thiazides, *n* (%)	20 (20.4)	30 (28.8)	25 (28.4)	39 (41.1)	.003

Data are given as *n* (%) or median (IQR)

BMI, body mass index; bpm, beats per minute; CKD, chronic kidney disease; eGFR, estimated glomerular filtration rate; HF, heart failure; IHD, ischaemic heart disease; LVEF, left ventricular ejection fraction; MRA, mineralocorticoid receptor antagonist; NT-proBNP, aminoterminal pro-brain natriuretic peptide; NYHA, New York Heart Association; RASi, renin-angiotensin-system inhibitors; TAPSE, tricuspid annular plane systolic excursion.

### Heatmap

The Spearman correlation heatmap coefficients are displayed in Supplementary Fig. S1. Total, intravascular, and tissue fluid excess had the strongest correlation with CCS (total fluid excess rho = 0.31; *P* < .001, intravascular fluid excess rho = 0.28; *P* < .001, tissue fluid excess rho = 0.33; *P* < .001), followed by NT-proBNP (tissue fluid excess rho = 0.24; *P* < .001, total fluid excess rho = 0.20; *P* < .001).

### Clinical endpoints

#### Outcomes according to bioimpedance analysis-derived estimates of fluid overload at baseline

The cumulative incidence of the composite outcome, its components, and all-cause mortality, according to quartiles of BIA-derived estimates of fluid overload at baseline, are shown in *Table 2*.

**Table 2:** Association between bioimpedance-derived fluid excess compartments and clinical outcomes

	Crude rate per 100-persons/year	Unadjusted HR (95% CI)	*P* value	Adjusted HR (95% CI)	*P* value
All-cause death or worsening HF event					
Total fluid excess quartiles					
Q1: <0.5	18.4 (11.2–30.0)	1.00 (Ref.)		1.00 (Ref.)	
Q2: 0.5 to 1.3	21.7 (14.0–33.6)	1.17 (0.61–2.27)	.624	1.04 (0.52–2.06)	.911
Q3: 1.4 to 2.3	26.3 (16.7–41.1)	1.39 (0.72–2.70)	.330	1.01 (0.51–2.01)	.960
Q4: >2.3	83.4 (62.5–111.4)	3.82 (2.16–6.76)	<.001	2.21 (1.21–4.05)	.010
Tissue fluid excess quartiles					
Q1: <1.1	15.8 (9.4–26.7)	1.00 (Ref.)		1.00 (Ref.)	
Q2: 1.1–1.9	23.1 (14.9–35.8)	1.43 (0.72–2.84)	.298	1.22 (0.79–3.16)	.577
Q3: 2.0–2.8	29.5 (19.6–44.4)	1.79 (0.92–3.49)	.084	1.58 (0.79–3.16)	.190
Q4: >2.8	82.0 (61.0–110.2)	4.29 (2.34–7.84)	<.001	2.81 (1.49–5.32)	.001
Intravascular fluid excess quartiles					
Q1: <1.1	23.4 (15.1–36.3)	1.00 (Ref.)		1.00 (Ref.)	
Q2: 1.1–1.9	18.8 (11.5–30.7)	0.81 (0.42–1.56)	.529	0.79 (0.40–1.53)	.480
Q3: 2.0–2.8	35.9 (24.6–52.3)	1.46 (0.82–2.61)	.194	1.27 (0.70–2.31)	.426
Q4: >2.8	62.1 (45.2–85.4)	2.35 (1.37–4.05)	.002	2.16 (1.19–3.90)	.011
Worsening HF events					
Total fluid excess quartiles					
Q1: <0.5	16.0 (9.5–27.1)	1.00 (Ref.)		1.00 (Ref.)	
Q2: 0.5 to 1.3	18.4 (11.5–29.6)	1.15 (0.57–2.28)	.690	0.94 (0.44–2.01)	.877
Q3: 1.4 to 2.3	17.5 (10.1–30.0)	1.06 (0.50–2.23)	.877	0.73 (0.34–1.52)	.400
Q4: >2.3	58.7.4 (42.3–81.3)	3.18 (1.75–5.81)	<.001	1.47 (0.77–2.79)	.237
Tissue fluid excess quartiles					
Q1: <1.1	12.4 (6.9–22.4)	1.00 (Ref.)		1.00 (Ref.)	
Q2: 1.1–1.9	19.7 (12.2–31.7)	1.56 (0.74–3.25)	.235	1.19 (0.55–2.55)	.655
Q3: 2.0–2.8	18.6 (11.2–30.9)	1.46 (0.68–3.15)	.324	1.15 (0.50–2.61)	.739
Q4: >2.8	62.0 (44.9–85.5)	4.31 (2.24–8.28)	<.001	2.21 (1.15–4.28)	.018
Intravascular fluid excess quartiles					
Q1: <1.1	17.6 (10.6–29.2)	1.00 (Ref.)		1.00 (Ref.)	
Q2: 1.1–1.9	86.0 (8.8–26.0)	0.88 (0.43–1.81)	.728	0.76 (0.37–1.56)	.458
Q3: 2.0–2.8	25.2 (16.3–39.0)	1.40 (0.73–2.68)	.304	1.03 (0.52–2.03)	.929
Q4: >2.8	49.4 (34.9–69.8)	2.58 (1.42–4.67)	.002	1.82 (1.00–3.31)	.047
All-cause mortality					
Total fluid excess quartiles					
Q1: <0.5	5.3 (2.2–12.6)	1.00 (Ref.)		1.00 (Ref.)	
Q2: 0.5 to 1.3	4.9 (2.0–11.7)	0.93 (0.27–3.20)	.906	1.18 (0.32–4.38)	.805
Q3: 1.4 to 2.3	8.3 (3.9–17.3)	1.57 (0.50–4.9)	.437	1.33 (0.39–4.50)	.641
Q4: >2.3	14.7 (8.6–25.4)	2.80 (1.00–7.87)	.050	2.79 (0.88–8.84)	.079
Tissue fluid excess quartiles					
Q1: <1.1	5.3 (2.2–12.8)	1.00 (Ref.)		1.00 (Ref.)	
Q2: 1.1–1.9	7.4 (3.5–15.4)	1.4 (0.44–4.35)	.581	1.19 (0.36–3.93)	.773
Q3: 2.0–2.8	8.6 (4.3–17.2)	1.61 (0.53–4.94)	.399	1.34 (0.41–4.38)	.619
Q4: >2.8	11.3 (6.0–20.9)	2.11 (0.72–6.18)	.172	1.71 (0.53–5.52)	.368
Intravascular fluid excess quartiles					
Q1: <1.1	8.6 (4.3–17.2)	1.00 (Ref.)		1.00 (Ref.)	
Q2: 1.1–1.9	6.4 (2.9–14.1)	0.74 (0.26–2.13)	.575	0.67 (0.22–1.96)	.454
Q3: 2.0–2.8	8.7 (4.4–17.5)	1.01 (0.38–2.70)	.978	0.94 (0.34–2.62)	.912
Q4: >2.8	8.7 (4.3–17.4)	1.00 (0.38–2.69)	.988	1.01 (0.33–3.08)	.975

Crude incidence rates (per 100 person-years) and hazard ratios with 95% confidence intervals for (i) the composite endpoint of all-cause death or worsening HF event, (ii) worsening HF events, and (iii) all-cause mortality, across quartiles (Q1–Q4) of total, tissue, and intravascular fluid excess (cut-points shown). Unadjusted and adjusted HRs are presented, using Q1 as the reference category; corresponding *P*-values are shown.

CI, confidence interval; HF, heart failure; HR, hazard ratio.

At 1-year follow-up, 101 patients reached the composite endpoint of all-cause death or worsening HF. The rate of events (per 100 person-years) increased across quartiles of total, tissue, and intravascular fluid excess, as is shown in *Table 2* and Supplementary Fig. S2. The risk of the composite outcome and a worsening HF event was higher across quartile 4 relative to quartile 1. However, the risk of all-cause death alone did not significantly differ across quartiles (*Table 2*).

In multivariable survival analysis, the association of BIA-derived estimates of fluid overload at baseline with the composite of 1-year all-cause death or WHF showed a similar pattern, with higher risk among patients in the highest quartile of total, tissue, and intravascular fluid excess, respectively (*Table 2*). This association was primarily driven by a higher risk of WHF events in patients in quartile 4 compared to those in quartile 1, particularly in the tissue fluid excess component (*Table 2*). The BIA–derived estimates of fluid overload were not associated with a higher risk of all-cause death at 1-year.

**Figure 1:**
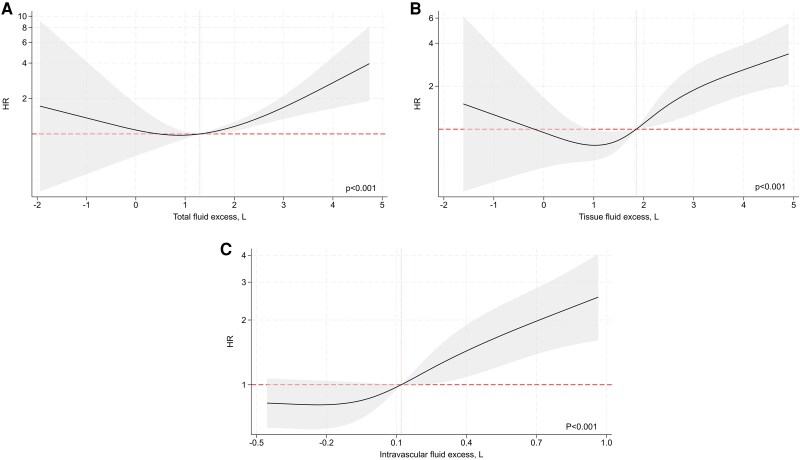
Association between bioimpedance-derived fluid excess—(*A*) total, (*B*) tissue, and (*C*) intravascular—and the 1-year risk of all-cause death or worsening heart failure. Each fluid compartment was modelled as a continuous exposure using restricted cubic splines in separate Cox models adjusted for age, composite congestion score, log N-terminal pro-B-type natriuretic peptide, blood urea nitrogen, plasma sodium, and treatment with renin-angiotensin-system inhibitors, aldosterone receptor antagonists, and sodium–glucose cotransporter 2 receptor inhibitors. The shaded areas represent 95% confidence intervals, and the vertical dashed lines denote the median value. HR, hazard ratio; BIA, bioimpedance; CCS, clinical congestion score; NT-proBNP, N-terminal pro-B-type natriuretic peptide; SGLT2, sodium–glucose cotransporter 2

**Figure 2:**
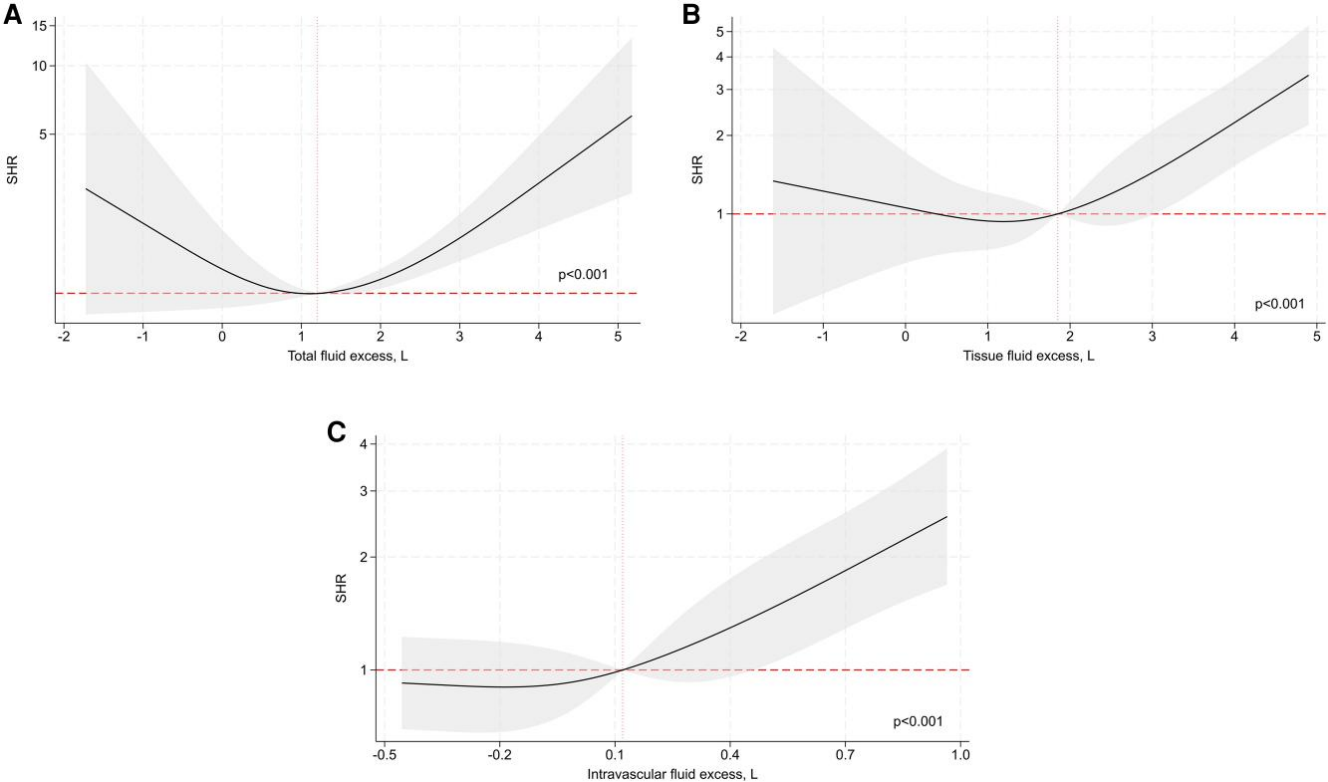
Association between bioimpedance-derived fluid excess—(*A*) total, (*B*) tissue, and (*C*) intravascular—and 1-year risk of worsening heart failure. Each fluid compartment was modelled as a continuous exposure using restricted cubic splines in separate Cox models adjusted for age, clinical congestion score, log N-terminal pro-B-type natriuretic peptide, blood urea nitrogen, plasma sodium, use of renin-angiotensin-system inhibitors, mineralocorticoid receptor antagonists, and sodium–glucose cotransporter 2 inhibitors, with all-cause death as a competing event. Shaded areas represent 95% confidence intervals; vertical dashed lines indicate median values. SHR, sub-hazard ratio; NT-proBNP, N-terminal pro-B-type natriuretic peptide; SGLT2, sodium–glucose cotransporter 2; BIA, bioimpedance; CCS, clinical congestion score

**Figure 3:**
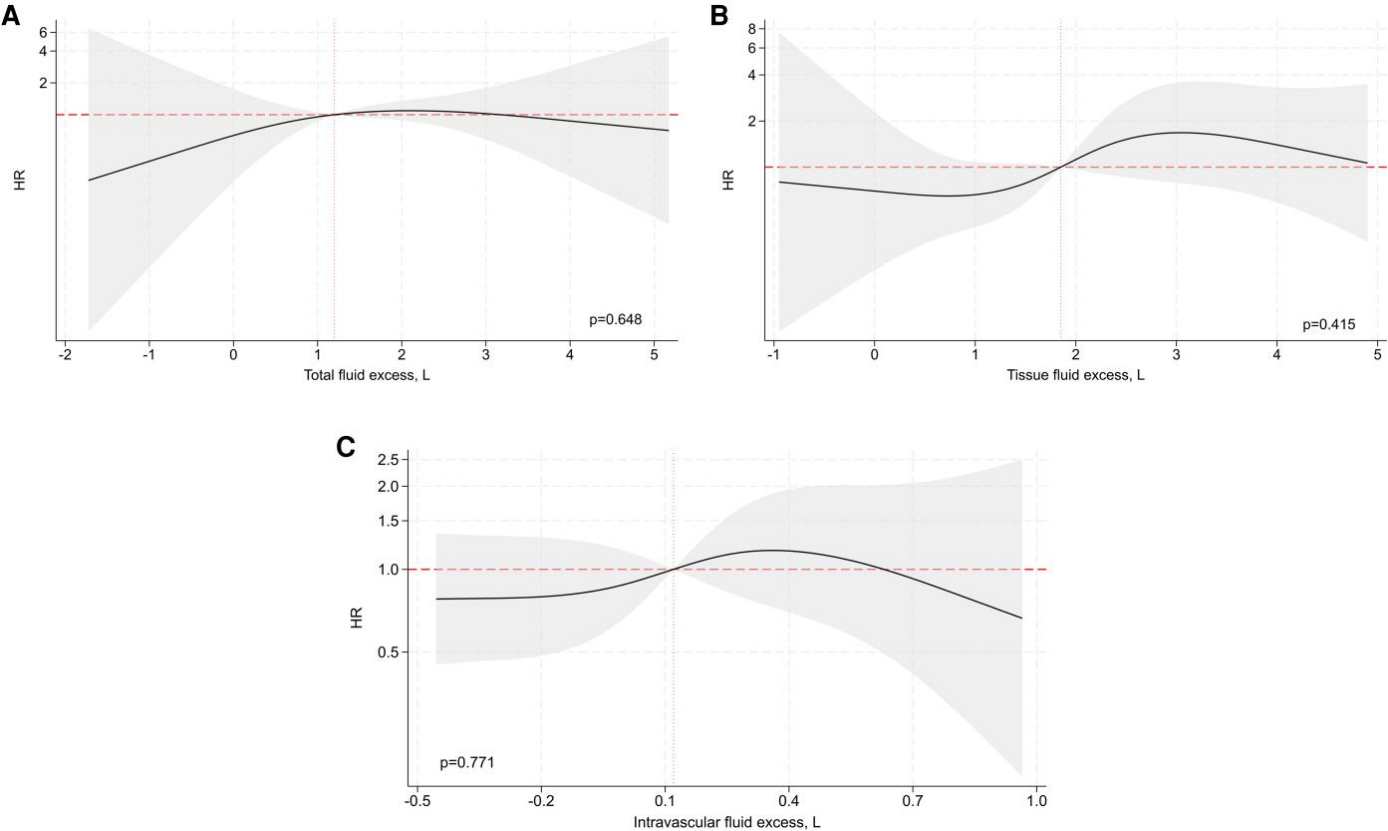
Association between bioimpedance-derived fluid excess—(*A*) total, (*B*) tissue, and (*C*) intravascular—and the 1-year risk of all-cause death. Each fluid compartment was modelled as a continuous exposure using restricted cubic splines in separate Cox models adjusted for age, composite congestion score, log N-terminal pro-B-type natriuretic peptide, blood urea nitrogen, plasma sodium, and treatment with renin-angiotensin-system inhibitors, aldosterone receptor antagonists, and sodium–glucose cotransporter 2 receptor inhibitors. The shaded areas represent 95% confidence intervals, and the vertical dashed lines denote the median value. HR, hazard ratio; NT-proBNP, N-terminal pro-B-type natriuretic peptide; SGLT2, sodium–glucose cotransporter 2; BIA, bioimpedance; CCS, clinical congestion score

The associations between BIA-derived estimates of fluid overload, analysed as continuous variables, and clinical outcomes are shown in *Figures 1–3*. In multivariable models, we observed significant non-linear associations between all fluid excess indices and the composite endpoint of WHF events or all-cause mortality. Total fluid excess showed a U-shaped relationship, with HRs below unity around values of 0–1 l but rising steeply at higher levels of overhydration (*Figure 1A*). A similar curvilinear pattern was observed for tissue fluid excess (*Figure 1B*). In contrast, intravascular fluid excess displayed a more monotonic association, with risk remaining stable at lower levels but increasing progressively above 0.1 l (*Figure 1C*). When worsening HF events were analysed separately, we found comparable patterns (*Figure 2*). By contrast, BIA-derived indices of fluid overload were not significantly associated with all-cause mortality (*Figure 3*).

#### Predictive value of bioimpedance analysis-derived estimates of fluid overload

**Figure 4:**
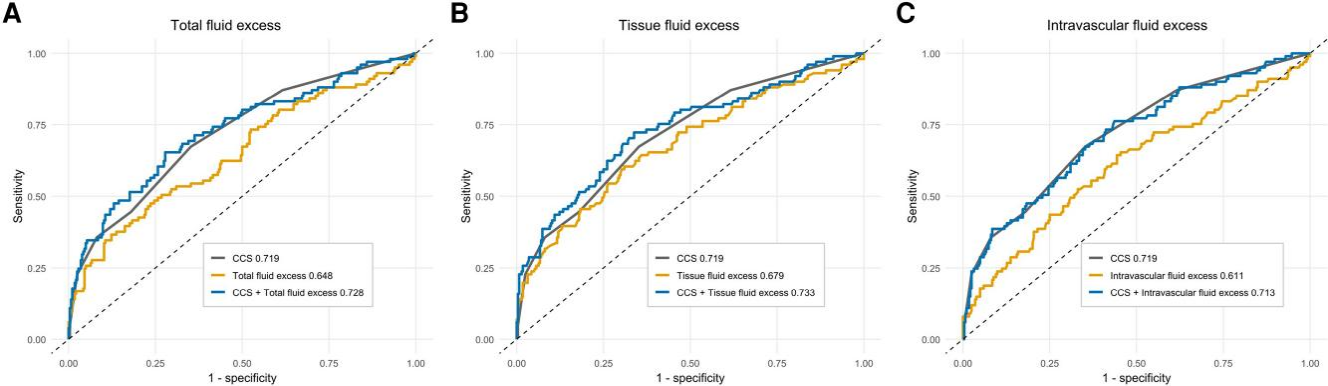
Receiver-operating characteristic curves for bioimpedance-derived measures of fluid excess—(*A*) total, (*B*) tissue, and (*C*) intravascular—in predicting the composite endpoint. The area under the curve is shown for each measure alone and when added to the clinical congestion score. BIA, bioimpedance; AUC, area under the curve; CCS, clinical congestion score

Tissue fluid excess had a relatively higher predictive value than total (AUC for the composite outcome: 0.679 vs 0.648; *P* = .023) and intravascular fluid excess (AUC for the composite outcome: 0.679 vs 0.611; *P* = .002). When individually added to the CCS model (AUC: 0.719) for the composite outcome, BIA-derived measures of fluid overload provided additional prognostic information (*Figure 4*). Adding tissue fluid excess resulted in a continuous NRI of 43.3% (95% CI: 22.6–64.1) and an integrated discrimination index (IDI) of 3.2% (95% CI: 1.1–5.4). Adding total fluid excess resulted in a continuous NRI of 30.0% (95% CI: 6.4–51.9) and an IDI of 2.4% (95% CI: 0.5–4.2). In contrast, adding intravascular fluid excess did not improve model discrimination [continuous NRI −3.0% (95% CI: −24.8 to 20.5); IDI 0.1% (95% CI: −0.2 to 0.5)]. In complementary analyses using either the MAGGIC score or NT-proBNP as baseline clinical models, adding BIA-derived fluid indices enhanced risk prediction, although the magnitude of improvement differed by compartment. Incorporating tissue fluid excess yielded the largest gain, with a continuous NRI of 48.4% (95% CI: 26.4–70.4) and an IDI of 7.3% (95% CI: 3.9–10.9) when added to MAGGIC, and an NRI of 49.0% (95% CI: 27.3–70.7) and an IDI of 6.4% (95% CI: 3.3–9.7) when added to NT-proBNP. Total fluid excess also improved classification, with NRI values of 41.4% (95% CI: 18.9–63.9) and 36.1% (95% CI: 13.6–58.6) and IDI gains of 6.2% (95% CI: 3.0–9.3) and 4.9% (95% CI: 2.2–7.7) on top of MAGGIC and NT-proBNP, respectively. In contrast, intravascular fluid excess improved risk reclassification (NRI ≈38%–41%) but did not meaningfully enhance discrimination (IDI ≈0%).

## Discussion

In this prospective cohort of ambulatory patients with chronic HF, bioimpedance-derived fluid overload was a significant predictor of adverse outcomes. Patients in the highest quartile of total, tissue, and intravascular fluid excess had significantly higher event rates, an association that remained significant after multivariable adjustment, including established prognostic markers, such as NT-proBNP and clinical surrogates of congestion. The excess risk was driven largely by worsening HF events, with no consistent association with all-cause mortality. When compartmental fluid excess was analysed as a continuous variable, a U-shaped association with risk emerged. For total and tissue fluid overload, the hazard was lowest around normal or slightly positive values, rising significantly in a dose-dependent manner with increasing excess. Conversely, a non-significant trend towards higher risk was also observed with negative fluid balance (i.e. dehydration). While this finding may reflect true clinical vulnerability related to hypovolemia, it should be interpreted cautiously, as measurement error or reverse causality in frailer patients with poor haemodynamic reserve could also contribute. In contrast, the associated risk for intravascular overload was lowest at a neutral or slightly negative balance, rising progressively and significantly with any degree of fluid excess. Among the three indices, tissue fluid excess provided the most clinically meaningful incremental prognostic information beyond CCS, NT-proBNP, and established risk models such as the MAGGIC score, whereas total and intravascular fluid excess, although associated with outcomes, contributed only limited additional value over conventional clinical assessment. Collectively, our results suggest that bioimpedance-derived compartmental measures of fluid overload offer incremental prognostic information and can refine the identification of patients vulnerable to near-term decompensation.

Our results support the growing consensus that BIA can be applied in diverse clinical scenarios to refine risk stratification.^9,12,13^ Previous studies in HF populations have reported that excess total and extracellular fluid burden, measured by different BIA/BIS devices, is an important predictor of mortality and hospital readmissions.^14–17^ This has led to renewed interest in whether serial BIA monitoring might help clinicians identify early, subclinical congestion before overt symptom worsening. While such approaches have gained more traction in nephrology (with large-scale observational data from the Fresenius Body Composition Monitor), evidence has also accumulated in HF cohorts that bioimpedance can detect ‘subclinical’ fluid overload.^18–20^ Studies using bioelectrical impedance vector analysis have shown that fluid excess, typically reflected by a rightward shift of the vector or elevated hydration indices, predicts both worsening renal function and long-term kidney disease progression in HF and chronic kidney disease cohorts.^21–23^ These observations highlight the close interplay between systemic congestion and renal vulnerability, supporting the concept that volume overload is not only a marker of haemodynamic stress but also a driver of adverse cardiorenal outcomes. However, there is substantial heterogeneity among BIA technologies, derived parameters, and modelling strategies, which complicates direct comparison across studies and partly explains discrepancies in prognostic strength between extracellular water (ECW) and total body water.^9^ Devices such as the BCM, which estimate relative fluid overload indexed to ECW via a three-compartment model, have consistently shown stronger and more reproducible prognostic associations, with validated risk thresholds (>7% moderate and >15% severe ECW overload) in both dialysis and non-dialysis settings (5). Against this background, our work extends the field by applying a novel BIA device (MALTRON® BioScan touch i8 IVF) capable of differentiating intravascular and tissue (extravascular) fluid compartments. This distinction appears clinically meaningful: we found that tissue fluid excess provided the strongest incremental prognostic value beyond clinical congestion assessment, NT-proBNP, and risk models such as the MAGGIC score. These findings support the concept that extravascular congestion is more closely linked to risk and underscore the need for standardized, compartment-specific BIA metrics in HF.

The present findings could enhance routine HF care by providing an objective, operator-independent measure of fluid status. Traditional reliance on physical examination or single biomarkers (e.g. NT-proBNP) is prone to variability and may not entirely capture fluid accumulation, especially in milder scenarios. Similar to prior work in both HF and advanced CKD,^9^ we propose that integrating BIA measurements into standard evaluations might be useful for uncovering subclinical congestion and prompt earlier diuretic adjustments, track trends in fluid distribution longitudinally, and support individualized volume management strategies. Such an approach may be especially relevant for older, multimorbid patients, in whom fluid management is often complicated by borderline renal function or neurohormonal dysregulation.^24,25^

Future investigations should consider several key areas: (i) whether repeated, longitudinal BIA assessments refine predictive accuracy over single ‘snapshot’ measurements; (ii) the optimal thresholds of tissue fluid excess in HF populations, which may differ from those derived in dialysis cohorts; and (iii) whether BIA-guided therapy confers clinically meaningful improvements—such as fewer hospitalizations or better quality of life—in a randomized setting. Recent trials in the dialysis population have suggested that ‘dry weight’ management guided by bioimpedance improves intermediate targets (e.g. blood pressure control), though definitive benefits on survival or cardiovascular events remain less clear.^26–28^ In HF, well-powered randomized studies are needed to confirm whether compartment-specific fluid assessment leads to improved outcomes beyond biomarker and symptom-based approaches. Finally, future work should explore the practical feasibility and economic implications of implementing routine BIA monitoring in HF care. Although BIA devices are generally portable and relatively inexpensive compared with imaging-based congestion assessment, integration into clinical pathways, and cost-effectiveness across different care settings remain largely untested.

### Limitations

First, this is an observational single-centre study of prospectively collected data from one tertiary HF clinic, which may limit broader applicability. Although we incorporated robust covariate adjustment, residual confounding cannot be excluded, and no causal inferences can be definitively drawn. Second, we specifically enrolled ambulatory patients. Consequently, the results may not be generalizable to individuals with acutely decompensated HF or other high-risk groups. Third, the BioScan touch i8 IVF device relies on proprietary algorithms to partition fluid across body compartments. While this feature offers theoretical advantages, the external validity of our findings is limited—particularly in clinical settings that use simpler, whole-body bioimpedance devices to which our results may not directly apply. Larger, independent studies in more diverse populations, directly comparing this technology with widely adopted BIA devices, are needed to confirm accuracy, reproducibility, and clinical applicability. Fourth, fluid overload was measured only once, precluding the evaluation of dynamic changes over time. Repeated measurements could enhance understanding of whether trends in tissue-specific fluid accumulation improve risk reclassification and guide therapy. Finally, patients were not specifically stratified by CKD stage, and thresholds validated in dialysis populations were not applied. Therefore, these results should not be extrapolated to those with more advanced renal dysfunction, where fluid management and overload may differ substantially.

### Conclusions

In this ambulatory HF cohort, compartment-specific bioimpedance measurements demonstrated a positive correlation with standard congestion markers and added prognostic value for adverse clinical outcomes. By adapting established techniques from the dialysis and CKD fields to the HF setting, BIA may serve as a non-invasive, practical tool to optimize patient stratification and volume management in chronic HF.

## Supplementary Material

xvag002_Supplementary_Data

## Declarations

### Data Availability

The datasets analysed during the current study are available from the corresponding author on reasonable request.

### Pre-registered Clinical Trial Number

None supplied.
