# Supplementary figures and images for: Bioimpedance-derived compartmental fluid status and prognosis in chronic heart failure
Source: ESC Heart Fail. 2026 Jan 22;13(1):xvag002. doi: 10.1093/eschf/xvag002 (PMC13108313; doi:10.1093/eschf/xvag002)

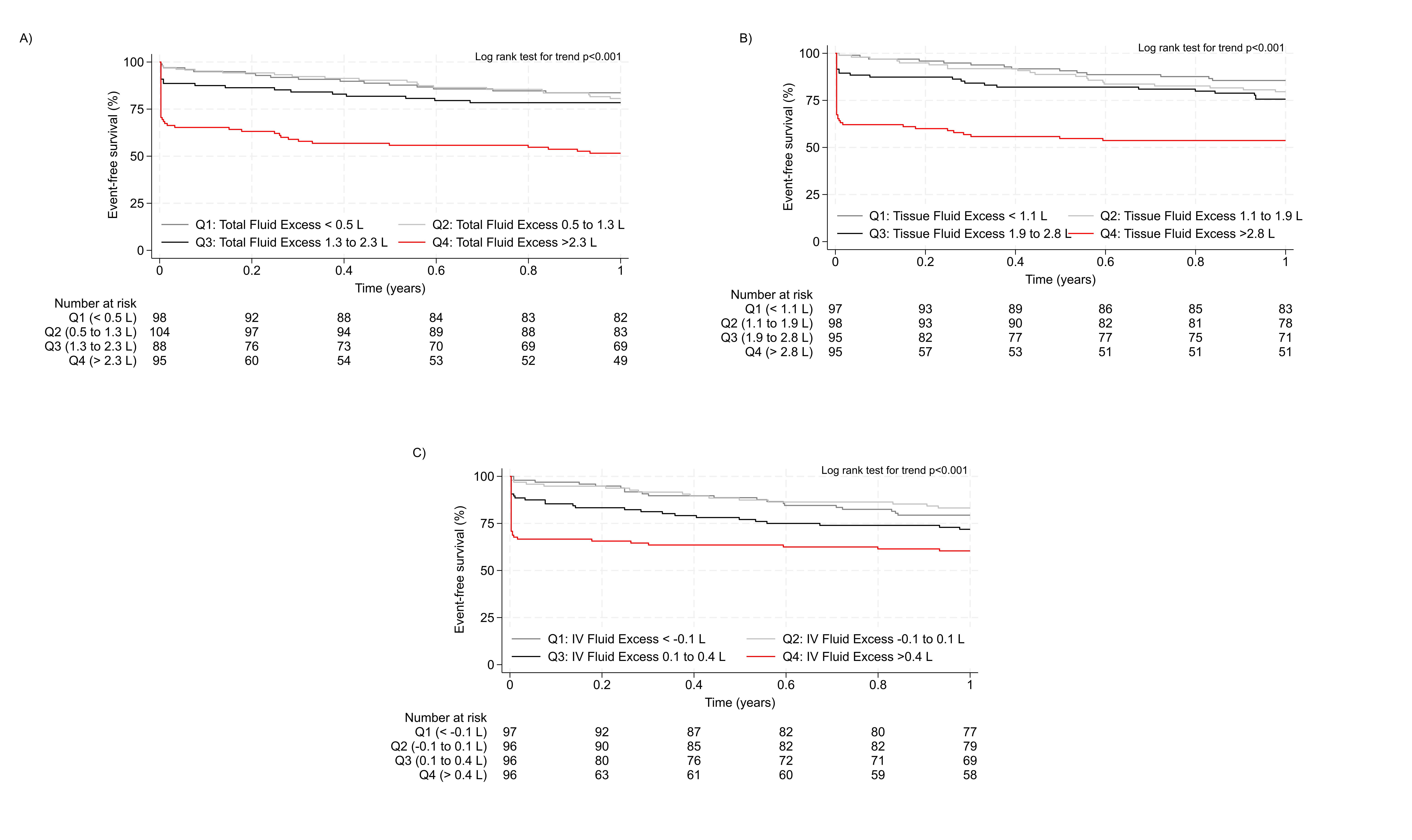

Supplement: xvag002_Supplementary_Data [file xvag002_supplementary_data.zip › Supp Figure 2.jpg]
